# Factors Affecting the Occupancy of Gaur (*Bos gaurus*) During Winter Season in Parsa National Park, Nepal

**DOI:** 10.1002/ece3.71189

**Published:** 2025-03-30

**Authors:** Bishnu Prasad Bhattarai, Hem Bahadur Katuwal, Sandeep Regmi, Bishnu Aryal, Krishna Tamang, Sabin KC, Amrit Nepali, Shivish Bhandari, Amir Basnet, Pradip Kandel, Chandu Paneru, Bishal Subedi, Niraj Regmi, Sabina Koirala, Haribhadra Acharya, Jerrold L. Belant, Hari Prasad Sharma

**Affiliations:** ^1^ Central Department of Zoology Institute of Science and Technology, Tribhuvan University Kirtipur, Kathmandu Nepal; ^2^ Nepal Zoological Society Kirtipur, Kathmandu Nepal; ^3^ Center for Integrative Conservation Xishuangbanna Tropical Botanical Garden, Chinese Academy of Sciences Mengla Yunnan China; ^4^ Natural Science Society Kirtipur, Kathmandu Nepal; ^5^ The Himalayan Conservancy Kirtipur, Kathmandu Nepal; ^6^ Key Laboratory of Ecological Safety and Sustainable Development in Arid Lands Xinjiang Institute of Ecology and Geography, Chinese Academy of Sciences Urumqi China; ^7^ Department of National Parks and Wildlife Conservation Babarmahal, Kathmandu Nepal; ^8^ Department of Fisheries and Wildlife Michigan State University East Lansing Michigan USA

**Keywords:** conservation, gaur, occupancy, Parsa National Park, predator, vulnerable

## Abstract

Gaur ( *Bos gaurus* ) is a globally vulnerable species with a decline of more than 80% of their global distribution in the past 100 years. Understanding the species distribution pattern and associated factors is essential for developing effective conservation strategies. We examined the effects of forest area, human detections, presence of tiger (*Panthera tigris*), presence of competing species like Asian elephant (*Elephas maximus*), and sambar deer (*Rusa unicolor*), and distance to water, on gaur occupancy in Parsa National Park (PNP), Nepal, using camera traps which were deployed at 67 locations from December 2022 to March 2023. We used single season single species occupancy modeling to estimate the relationship of selected covariates with gaur occupancy. We recorded a total of 54 gaur detections in our study. We found that gaur occupancy had a significant positive association with the distance to water bodies and was negatively associated with forest area and the presence of elephants; however, there was no significant association with number of humans detected, or the presence of tigers, or sambar deers. Gaur had greater detection probabilities in southcentral portions of PNP, i.e., flat plains and areas near the Chure region and the lowest detection probabilities in the eastern and western parts of PNP. These findings highlight the importance of considering eco‐environmental factors in the management and conservation of gaur, particularly in human‐dominated landscapes. We recommend further multi‐seasonal studies to better understand the dynamic interactions between gaur, their environment, and other species, to inform effective conservation strategies.

## Graphical abstract

Gaur occupancy was positively influenced by thedistance to waterbodies and negatively influenced by forest area and presence of elephants..

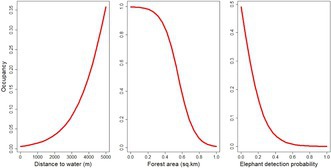

## 1 Introduction

Large mammalian herbivores occupy half of the Earth's terrestrial surface and play a critical role both ecologically and economically (Olff et al. 2002). Despite their importance, 60% of large herbivore species are currently listed as threatened with extinction by the International Union for the Conservation of Nature (Ripple et al. 2015). These herbivores have both direct and indirect impacts on ecosystem function, influencing plant community structure and composition (Tuomi et al. 2019) and contributing to nutrient cycling (Pringle et al. 2023). Their presence and behavior create cascading effects across trophic levels, shaping community dynamics and ecosystem function by altering primary productivity through top‐down processes (Pringle et al. 2023; Allred et al. 2011).

The largest wild bovid, Gaur ( *Bos gaurus* ), occurs globally and historically across southcentral and southeast Asia (Duckworth et al. 2016). The IUCN Red List of Threatened Species lists the gaur as vulnerable to extinction, and their global distribution has declined more than 80% in the past 100 years (Groves and Grubb 2011). The estimated global population of gaur is 15,000–35,000 individuals; however, its abundance is little understood in many countries (Duckworth et al. 2016).

Gaurs typically select grassland habitats and open areas within moist and dry evergreen forests, semi‐evergreen forests, and mixed deciduous forests (Bhumpakphan and McShea 2011). They are generally seen in groups, with each group consisting of around 5–12 individuals (Sankar et al. 2013; Menon 2023). Gaur habitats are generally characterized by vast, mostly undisturbed forested areas, hilly landscapes below 1800 m in elevation, proximity to water sources, and an abundant supply of coarse grasses (including bamboo), shrubs, and trees (Nowak 1999). While gaurs rely on water for drinking and bathing, they do not engage in wallowing behavior (Nowak 1999). The availability of water is a crucial factor for their habitat selection, and fluctuations in water availability can influence the spatio‐temporal distribution of the species (Bhattarai and Kindlmann 2013; Imam and Kushwaha 2013; Schmied née Stommel et al. 2024).

In addition to water availability, co‐occurring species also shape the habitat use of gaurs. For instance, tiger habitat use is increased by increasing the gaur occupancy (Sharma et al. 2025), and gaurs may shift their space use away from areas with a higher presence of predators (Karanth and Stith 1999). Similarly, larger herbivores such as Asian elephants ( *Elephas maximus* ) may affect gaur movement through direct or indirect competition, as observed between African elephants ( *Loxodonta africana* ) and smaller ungulates (Fritz et al. 2002). Human activities within gaur habitats, such as resource collection and recreational pursuits, pose additional threats, as gaurs are inherently shy animals (Choudhury 2002). These disturbances can disrupt their natural behavior, altering their movement and foraging patterns, which further increases their vulnerability to extinction.

In Nepal, gaurs face multiple threats, including habitat loss and fragmentation, poaching, and encroachment (DNPWC 2020). Despite their limited range and the serious conservation challenges they face, there has been relatively little research into the ecology of gaurs in Nepal. The National Parks and Wildlife Conservation Act 1973 in Nepal protects gaurs (DNPWC 2019). The estimated number of gaurs in Nepal ranged from 250 to 350 individuals in the mid‐1990s to about 473 individuals (368 in Chitwan National Park and 105 in Parsa National Park) in 2016, all of which are in protected areas (DNPWC 2019). In Chitwan National Park, the species population almost doubled between 1997 and 2016, from 188 individuals to 368 individuals (DNPWC 2019). Due to their shy nature (Choudhury ) and their preference for the core areas of protected regions (Zangmo et al. 2018), limited information is available about their behavior and habitat use.

This study aims to understand the factors influencing gaur occupancy in Parsa National Park (PNP), Nepal. We hypothesized that gaur occupancy would decrease in areas with higher tiger presence due to predation risk and with the presence of large herbivores due to competition for forage. Additionally, we expected gaur occupancy to increase with greater forest cover and decrease with increasing distance from water sources. We also anticipated a decline in gaur occupancy in areas with higher levels of human activity.

## 2 Material and Methods

### 2.1 Study Area

**FIGURE 1:**
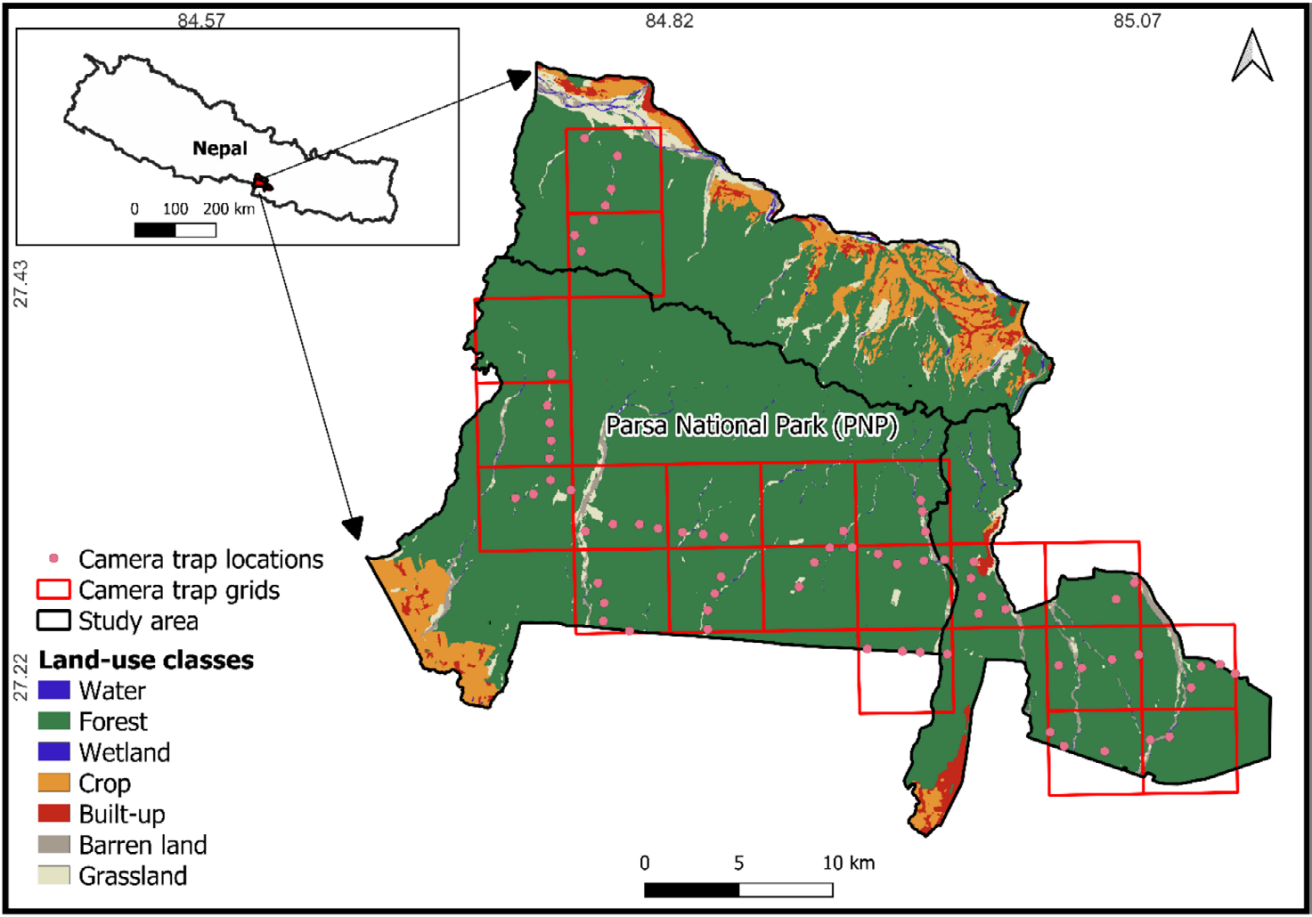
Study areas for gaur in Parsa National Park, Nepal, overlaid with 5 km × 5 km study grids.

This study was conducted in Parsa National Park (PNP), Nepal (Figure 1). Located in the subtropical zone of southern Nepal, PNP, and its buffer zone cover a total area of 912.17 km^2^ (Park: 627 km^2^, buffer zone: 285.17 km^2^). The park encompasses the three districts, Makawanpur, Parsa, and Bara in the lowland of Nepal, with elevations ranging from 435 to 950 m above sea level.

The PNP encompasses parts of the Tarai region and the Chure Hills of Nepal. The area experiences four distinct seasons: summer (April–June), monsoon (July–September), winter (October–December), and spring (January–March). The park's vegetation is dominated by tropical and subtropical species, including Sal ( *Shorea robusta* ) forests, riverine forests with species such as sissoo (*Dalbergia sisoo*), silk cotton tree ( *Bombax ceiba* ), and khair ( *Acacia catechu* ), as well as grasslands featuring siru ( *Imperata cylindrica* ) and kans ( *Saccharum spontaneum* ).

The PNP serves as a critical habitat for several threatened wildlife species, including the Asian elephant ( *Elephas maximus* ), tiger ( *Panthera tigris* ), leopard ( *P. pardus* ), and sloth bear ( *Melursus ursinus* ) (Sharma et al. 2023). The park also supports a diverse range of other mammal species, such as the blue bull ( *Boselaphus tragocamelus* ), sambar deer ( *Rusa unicolor* ), hog deer ( *Axis porcinus* ), northern red muntjac (*Muntiacus vaginalis*), and rhesus macaque ( *Macaca mulatta* ). Despite its ecological importance, PNP faces significant anthropogenic pressures, including the collection of firewood and fodder, as well as cattle grazing (DNPWC 2020).

### 2.2 Data Collection

We collected data between December 2022 and March 2023 using remote camera traps (Stealth Cam‐STCG45NG) deployed at 67 locations, ensuring a minimum distance of 1 km between adjacent cameras. We designed a 5 km × 5 km grid size and deployed four cameras within a grid. Areas with human settlements and farmland were excluded (Figure 1), as the study species inhabit the core habitats of PNP. The cameras were placed along walking trails, with each camera having a range of approximately 20 m and an angle coverage of 41.3° field of view. The camera array covered a convex hull of ~659 km^2^ across PNP. Cameras were positioned 40–60 cm above the ground along fire‐lines and trails, and each was programmed to capture three images with a 30‐s delay before the next burst of three images. Cameras were checked weekly for 3 weeks before being relocated to a new site. Over the study period, the cameras were operational for a total of 1407 trap nights (67 sites for 21 days). Areas with difficult terrain and steep slopes, particularly on the northern side of the park where gaurs were not observed, were excluded from the study.

At each camera location, we recorded the distance to the nearest permanent water body, the area of forest within a 500‐m radius, the number of humans detected, and the presence of tigers, elephants, and sambar deer. These variables were used as covariates in the analysis of gaur occupancy. The distance to permanent water bodies was measured using the QGIS measure line tool, and the forest area was calculated using zonal statistics. Water bodies that retained water year‐round were classified as permanent. From the camera images, we recorded all detections of gaur, tiger, human, elephant, and sambar.

As an ethical consideration, we informed local communities and park officials about the camera trap deployment before installation. We ensured that any images capturing human presence would be used exclusively for scientific analysis and not for any other purposes. This transparency helped to build trust and respect within the community, upholding ethical research practices.

### 2.3 Data Analysis

We first performed correlation analyses for continuous variables using a threshold of |*r*| > 0.70 (Dormann et al. 2013). No variables were highly correlated (|*r*| ≤ 0.70), and we used all for the analysis. We used hierarchical occupancy modeling developed by Royle and Dorazio (2008) following Regmi et al. (2023) and Sharma et al. (2023) in the R program (R Core Team 2023) to assess detection probability, occupancy, and impacts of covariates on Gaur. We used each week of 21 days (total survey duration) as a single sampling occasion representing three replicate surveys. We created the object data as a matrix of species detections at each site, i.e., where the matrix comprised the number of detections for each sampling replicate. We used occupancy as an indicator of habitat selection rather than using true occupancy (Sunarto et al. 2012; Gould et al. 2019).

We derived occupancy as:

$$z_{i}\sim \text{Bernoulli}\left(\psi \right)$$

where *z* is a latent variable inferred from detection histories. *z*_*i*_ is drawn from a Bernoulli distribution with a parameter of probability *ψ*. We then modeled detection probability using a binomial distribution where *z*_*i*_ = 1, *p* is the probability of success and *z*_*i*_ = 1, the probability of success = 0 (*y*_*i*_ ~ Binomial (*n*_*i*_, *pz*_*i*_)). Where *i* is the number of sites and *ni* is the number of replicates out of the total when the species is detected at each site *i*.

We employed hierarchical occupancy modeling to assess the influence of various factors on the occurrence of gaur. We used logistic regression to determine the effect of hypothesized ecological covariates on gaur occurrence.

Since *ψ* is a probability of occupancy, the equation is given as,

$$\text{logit}\;\left(\psi_{i}\right)=\beta_{0}+\beta_{forest}+\beta_{\text{water}}+\beta_{tiger}+\beta_{\text{elephant}}+\beta_{sambar}+\beta_{human}$$

where, *β*_0_ = *logit*(*ψ*_*0*_) and *β* varies for each species. Unlike the previous model, we incorporated correlation between detection probability and occupancy and intercept. *β*_0_ is the probability of occupancy of the species at site i with a given combination of variables (Devarajan et al. 2020). We calculated mean estimated detection probability as the mean of observation probability across the study sites. For detection probability we assumed that each site has equal probability of detecting the species.

We generated model output using MCMC simulation and confirmed model convergence using Rhat values (Gelman‐Rubin statistic) with a threshold of 1.10. We ran adaptive MCMC simulations using the jagsUI (Kellner et al. 2022) and coda (Plummer et al. 2006) packages in R program (R Core Team 2023) and Just Another Gibbs Sampler (JAGS; Plummer et al. 2006) with 1000 adaptations, three chains, and 10,000 iterations with 1000 burn ins (Andrieu and Thoms 2008).

We created a detection probability map (1‐km resolution) of gaur across PNP using inverse distance weighting (IDW) interpolation in QGIS with the tool “IDW Interpolation” (QGIS Development Team 2023). The attribute for interpolation was the detection probability of the species across the 67 sites. Detection probabilities for each site were derived as a proportion of survey replicates in which the species is detected.

## 3 Results

We recorded a total of 277 carnivore detections, including 182 tiger detections, 63 Asian elephant detections and 54 gaur detections across the study sites. The mean forest area (+ standard deviation) across the sites was 0.733 ± 0.108 km^2^, and the mean distance to the nearest permanent water source was 3526.2 ± 2578.5 m. On average, the number of human detections per site was 90.267 ± 362.353. The mean detection probability for tigers was 0.657 ± 0.478, for elephants 0.239 ± 0.430, and for sambar 0.403 ± 0.494. The estimated mean detection probability for gaur was 0.400 ± 0.094, and the mean estimated occupancy was 0.308 ± 0.067.

**FIGURE 2:**
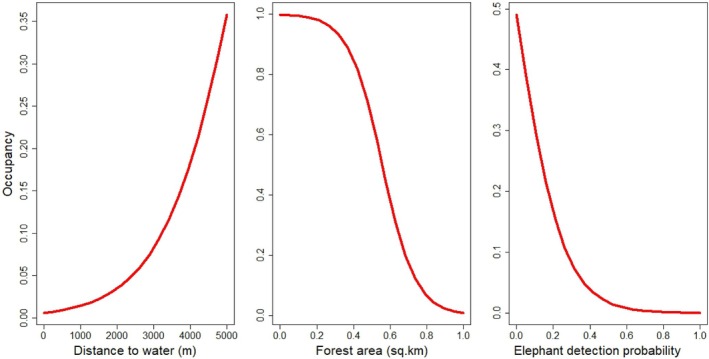
Effect of distance to water, forest area, and elephant on gaur occupancy in Parsa National Park, Nepal, December 2022–March 2023.

**FIGURE 3:**
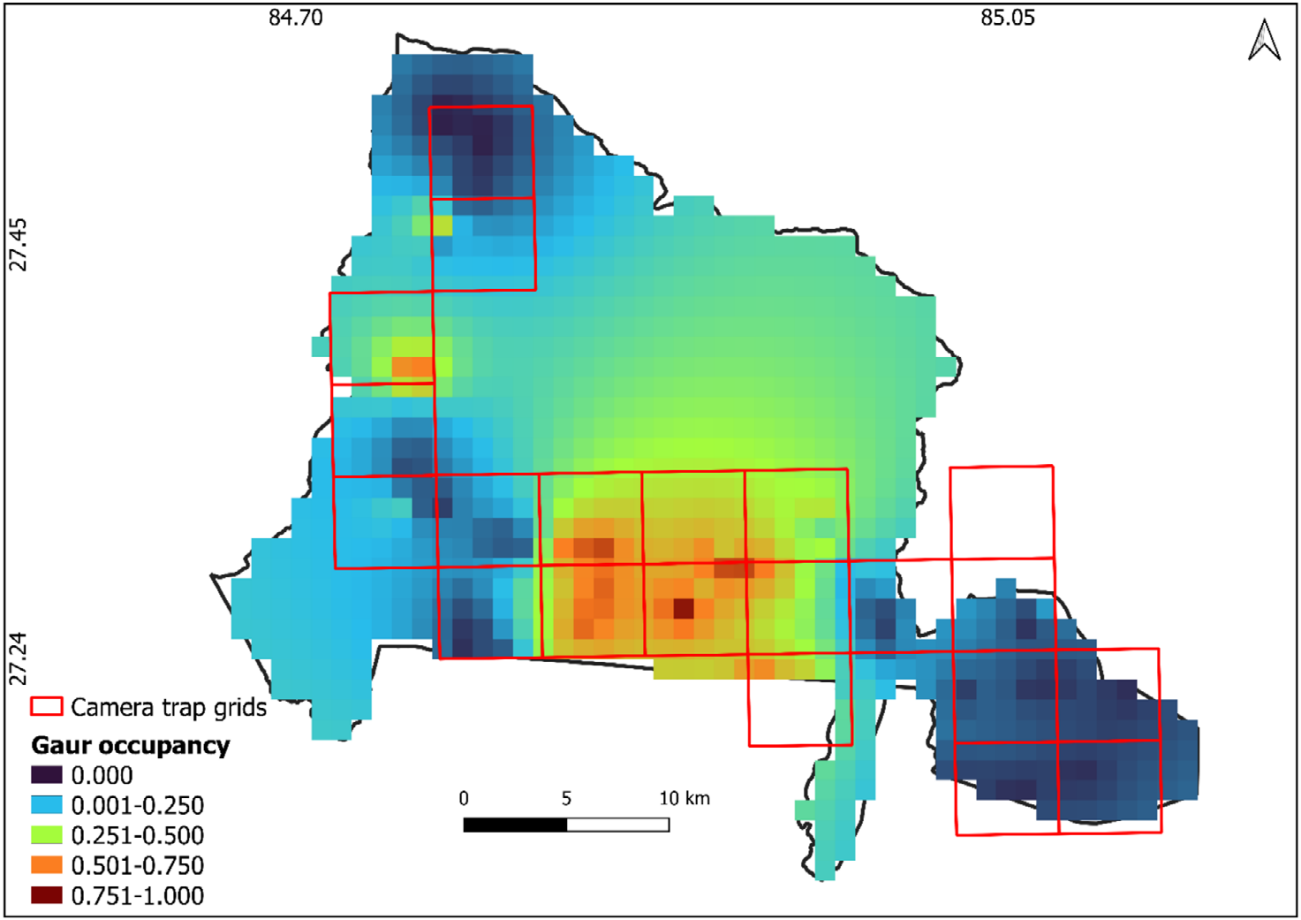
Occupancy probability (probability of habitat use) of gaur in Parsa National Park, Nepal, December 2022–March 2023.

Gaur occupancy had a significant positive relation with the nearest distance from water bodies (2.354 ± 0.815), while it decreased with increasing forest area (−1.185 ± 0.801) and the presence of elephants (−3.403 ± 1.108; Table 1, Figure 2). No significant relationship was observed between gaur occupancy and the number of humans detected (−1.215 ± 1.991), tiger presence (1.275 ± 1.352), or sambar presence (0.196 ± 1.307). The southcentral regions of PNP, where higher gaur occupancy was observed, are characterized by flat plains, a mix of grasslands and forests, and lower human disturbance, providing optimal foraging and movement habitats for gaur. These areas offer a more suitable environment for gaur compared to other parts of the park (Figure 3).

**TABLE 1:** Effect of covariates on gaur occupancy in Parsa National Park, Nepal, December 2022–March 2023.

Parameter	Mean	SD	LCI	UCI	Rhat	Overlap 0
b0 (intercept)	−1.930	1.176	−4.290	0.370	1.000	1
Distance to water (m)	**2.354**	**0.815**	**0.980**	**4.209**	**1.000**	**0**
Forest area (km^2^)	**−1.185**	**0.801**	**−3.274**	**−0.055**	**1.000**	**0**
Number of humans (*n*)	−1.215	1.991	−4.600	3.826	1.000	1
Tiger presence	1.275	1.352	−1.329	4.033	1.000	1
Elephant presence	**−3.403**	**1.108**	**−4.931**	**−0.946**	**1.000**	**0**
Sambar presence	0.196	1.307	−2.521	2.710	1.000	1

*Note:* Values in bold represent the significant association (credible intervals did not overlap at 0).

Abbreviations:SD, standard deviation; LCI, lower credible interval; UCI, upper credible interval.

## 4 Discussion

Our study found that gaur occupancy in Parsa National Park was positively associated with distance from water bodies, potentially due to reduced competition and predation risk near water sources. In contrast, forest area and the presence of elephants negatively affected gaur presence, likely due to limited access to forage in dense forests and competition with elephants. Surprisingly, human presence, tigers, and sambar had no significant impact on gaur occupancy, suggesting that human activities may be non‐intrusive or that gaur have adapted to certain disturbances. However, these results should be interpreted with caution, as finer‐scale habitat assessments and long‐term monitoring could reveal more nuanced ecological interactions influencing gaur distribution. We observed that gaurs were restricted to certain areas near the Chure region and flat plains of PNP. This pattern aligns with previous studies, which have reported the species' primary occurrence in the Tarai and Siwalik regions of southern Nepal. However, gaur populations are now limited to specific pockets within Chitwan and Parsa National Parks (Chetri 2003). This restricted distribution may explain the limited gaur presence in our study area. Identifying key hotspots of gaur occurrence is critical for wildlife habitat management, particularly in human‐dominated landscapes such as the Terai Arc Landscape, where large herbivores are confined to smaller protected areas (Wikramanayake et al. 2011).

Contrary to our initial hypothesis, we found a positive association between gaur occupancy and distance from the nearest permanent water body. Additionally, gaur may prefer habitats farther from water bodies due to better forage availability in forest edges and open grasslands, which provide high‐quality grazing opportunities. Furthermore, human activities such as fishing, livestock grazing, or tourism are often concentrated near water sources, potentially leading to localized disturbances that influence gaur habitat selection. Moreover, this could be attributed to the dry winter season during our study period and the potential presence of competitors or predators near water sources (Makin et al. 2017). Although mammal species are generally more concentrated near water bodies, especially herbivores during the dry season (Leweri et al. 2022; Rich et al. 2016), our results suggest that gaurs may avoid waterholes frequented by elephants. While previous studies have shown that gaurs tend to select habitats near water (Bhattarai and Kindlmann 2013; Imam and Kushwaha 2013), some reports, such as Paliwal and Mathur (2012), have similarly observed gaurs at considerable distances from water sources. It is likely that during the dry season, gaurs may alter their behavior to avoid areas heavily used by elephants (Valeix et al. 2007). These factors highlight the complex ecological dynamics shaping gaur distribution and warrant further investigation for effective conservation management.

In contrast to our expectations, we observed a negative association between forest area and gaur occupancy. Although many studies have reported a positive relationship between gaurs and forest cover (Goswami 2007; Imam and Kushwaha 2013), our results may reflect the gaurs' preference for grassland habitats during the dry season (Sankar et al. 2013). This behavior aligns with observations from Prayoon et al. (2024), who found that gaurs preferred habitats with lower canopy cover in Khao Phaeng Ma Non‐Hunting Area, Thailand. Other studies have also indicated that gaurs exhibit a seasonal preference for grasslands, despite their use of forested areas at other times (Bhumpakphan and McShea 2011; Sankar et al. 2013).

The negative impact of elephant presence on gaur occupancy in our study is consistent with the competitive interactions often observed between large herbivores. Elephants, as mega‐herbivores weighing over 1000 kg, consume large quantities of vegetation daily (Owen‐Smith 1988). Similar to the competitive effects reported between African elephants and other browsing herbivores (Fritz et al. 2002), competition may also exist between Asian elephants and gaurs (Steinheim et al. 2005). Although gaurs are primarily grazers, they occasionally switch to browsing (Ahrestani et al. 2012; Haleem and Ilyas 2018), and during the dry season, competition for resources with elephants may intensify. This competitive dynamic was also observed in Hluhluwe Game Reserve, South Africa, where a reduction in elephant populations corresponded with increased browsing by other herbivores (Owen‐Smith 1988; Steinheim et al. 2005). Additionally, the larger size and aggressive behavior of elephants may cause gaurs to avoid areas close to them (King 1973). Similarly, the insignificant associations with tigers and sambar might be attributed to substantial prey density and spatial segregation in habitat use.

## 5 Conclusions

Our study on gaur occupancy in Parsa National Park reveals that gaur occupancy was positively associated with increasing distance from water bodies, likely reflecting behavioral adaptations during the dry season to avoid areas frequented by larger competitors such as elephants. Conversely, forest area and elephant presence negatively impacted gaur occupancy, highlighting possible competition for resources. Unexpectedly, human presence, tigers, and sambar deer had no significant effect on gaur occupancy. These findings suggest that gaur prefer certain grassland habitats over forested areas during the dry season and avoid areas near elephants, possibly due to competition or avoidance of aggressive interactions. Our short‐term study results might be valuable for conservation and management strategies, particularly in human‐dominated landscapes like the Terai Arc Landscape, where large herbivores are confined to smaller protected areas. Identifying and preserving key hotspots for gaur will be essential to their long‐term survival. We acknowledged that informing local communities about camera deployments may have led to reduced human activity near the camera traps, introducing a potential bias. However, given the short duration of our study during the dry season, the influence of this bias on detection rates is limited and warrants further exploration in future multi‐seasonal studies.

## Author Contributions

**Bishnu Prasad Bhattarai:** conceptualization (equal), funding acquisition (lead), data curation (equal), investigation (equal), methodology (equal), writing – original draft (equal), writing – review and editing (equal). **Hem Bahadur Katuwal:** conceptualization (equal), funding acquisition (lead), data curation (equal), formal analysis (equal), investigation (equal), methodology (equal), writing – original draft (equal), writing – review and editing (equal). **Sandeep Regmi:** data curation (equal), formal analysis (equal), investigation (equal), methodology (equal), writing – original draft (equal), writing – review and editing (equal). **Bishnu Aryal:** investigation (equal), writing – review and editing (equal). **Krishna Tamang:** investigation (equal), writing – review and editing (equal). **Sabin KC:** investigation (equal), writing – review and editing (equal). **Amrit Nepali:** investigation (equal), writing – review and editing (equal). **Shivish Bhandari:** investigation (equal), methodology (equal), writing – original draft (equal), writing – review and editing (equal). **Amir Basnet:** investigation (equal), writing – review and editing (equal). **Pradip Kandel:** data curation (equal), writing – review and editing (equal). **Chandu Paneru:** data curation (equal), writing – review and editing (equal). **Bishal Subedi:** data curation (equal), writing – review and editing (equal). **Niraj Regmi:** data curation (equal), writing – review and editing (equal). **Sabina Koirala:** funding acquisition (lead), writing – review and editing (equal). **Haribhadra Acharya:** writing – review and editing (equal). **Jerrold L. Belant:** writing – review and editing (equal). **Hari Prasad Sharma:** conceptualization (equal), data curation (equal), formal analysis (equal), funding acquisition (lead), investigation (equal), methodology (equal), project administration (equal), validation (equal), visualization (equal), writing – original draft (equal), writing – review and editing (equal).

## Ethics Statement

Camera traps were deployed within the protected area, and necessary permissions were obtained from the Department of National Parks and Wildlife Conservation (Permission Number: 1165/2079‐80). People were informed about the camera traps before deploying.

## Conflicts of Interest

The authors declare no conflicts of interest.

## Data Availability

Data are available in Dryad: https://doi.org/10.5061/dryad.t4b8gtjb8. http://datadryad.org/stash/share/U3rU5vUV_4j6Z6TWGNwX‐TYQwKDomIUKTShVmkby6AY.

## References

[ece371189-bib-0001] Ahrestani, F. S. , I. M. A. Heitkönig , and H. H. T. Prins . 2012. “Diet and Habitat‐Niche Relationships Within an Assemblage of Large Herbivores in a Seasonal Tropical Forest.” Journal of Tropical Ecology 28, no. 4: 385–394.

[ece371189-bib-0002] Allred, B. W. , S. D. Fuhlendorf , and R. G. Hamilton . 2011. “The Role of Herbivores in Great Plains Conservation: Comparative Ecology of Bison and Cattle.” Ecosphere 2, no. 3: 1–17.

[ece371189-bib-0003] Andrieu, C. , and J. Thoms . 2008. “A Tutorial on Adaptive MCMC.” Statistics and Computing 18, no. 4: 343–373.

[ece371189-bib-0004] Bhattarai, B. P. , and P. Kindlmann . 2013. “Effect of Human Disturbance on the Prey of Tigers in the Chitwan National Park—Implications for Park Management.” Journal of Environmental Management 131: 343–350. 24211382 10.1016/j.jenvman.2013.10.005

[ece371189-bib-0005] Bhumpakphan, N. , and W. J. McShea . 2011. “Ecology of Gaur and Banteng in the Seasonally Dry Forests of Thailand.” In The Ecology and Conservation of Seasonally Dry Forests in Asia, edited by W. J. McShea , 165–178.

[ece371189-bib-0006] Chetri, M. 2003. “Food Habits of Gaur *Bos gaurus gaurus* and Livestock in Parsa Wildlife Reserve, Central Nepal.” Himalayan Journal of Sciences 1, no. 1: 31–36.

[ece371189-bib-0007] Choudhury, A. 2002. “Distribution and Conservation of the Gaur *Bos gaurus* in the Indian Subcontinent.” Mammal Review 32, no. 3: 199–226.

[ece371189-bib-0008] Devarajan, K. , T. L. Morelli , and S. Tenan . 2020. “Multi‐Species Occupancy Models: Review, Roadmap, and Recommendations.” Ecography 43, no. 11: 1612–1624.

[ece371189-bib-0009] DNPWC . 2020. “Conservation Action Plan of Gaur for Nepal (2020–2024).” Department of National Parks and Wildlife Conservation, Kathmandu, Nepal.

[ece371189-bib-0010] Dormann, C. F. , J. Elith , S. Bacher , et al. 2013. “Collinearity: A Review of Methods to Deal With It and a Simulation Study Evaluating Their Performance.” Ecography 36, no. 1: 27–46. 10.1111/j.1600-0587.2012.07348.x.

[ece371189-bib-1001] Duckworth, J. W. , K. Sankar , A. C. Williams , N. S. Kumar N , and R. J. Timmins . 2016. “*Bos gaurus*.” The IUCN Red List of Threatened Species 2016: e. T2891A46363646.

[ece371189-bib-0011] Fritz, H. , P. Duncan , I. J. Gordon , and A. W. Illius . 2002. “Megaherbivores Influence Trophic Guilds Structure in African Ungulate Communities.” Oecologia 131, no. 4: 620–625. 28547558 10.1007/s00442-002-0919-3

[ece371189-bib-0012] Goswami, K. G. 2007. “Habitat Preference of Indian Bison (*Bos gaurus*) During Summer in Billigiri Rangaswamy Temple Wildlife Sanctuary, Karnataka, India.” Nature, Environment and Pollution Technology 6, no. 1: 117–120.

[ece371189-bib-0013] Gould, M. J. , W. R. Gould , J. W. Cain , and G. W. Roemer . 2019. “Validating the Performance of Occupancy Models for Estimating Habitat Use and Predicting the Distribution of Highly Mobile Species: A Case Study Using the American Black Bear.” Biological Conservation 234: 28–36. 10.1016/j.biocon.2019.03.010.

[ece371189-bib-1002] Groves, C. , and P. Grubb . 2011. Ungulate Taxonomy, 317. Johns Hopkins University Press.

[ece371189-bib-1006] Haleem, A. , and O. Ilyas . 2018. “Food and Feeding Habits of Gaur (*Bos gaurus*) in Highlands of Central India: A Case Study at Pench Tiger Reserve, Madhya Pradesh (India).” Zoological Science 35: 57–67. 29417898 10.2108/zs170097

[ece371189-bib-0014] Imam, E. , and S. P. S. Kushwaha . 2013. “Habitat Suitability Modelling for Gaur ( *Bos gaurus* ) Using Multiple Logistic Regression, Remote Sensing, and GIS.” Journal of Applied Animal Research 41, no. 2: 189–199.

[ece371189-bib-0015] Karanth, K. U. , and B. M. Stith . 1999. “Prey Depletion as a Critical Determinant of Tiger Population Viability.” In Riding the Tiger: Tiger Conservation in Human‐Dominated Landscapes, edited by J. Seidensticker , S. Christie , and P. Jackson , 100–113.

[ece371189-bib-0016] Kellner, K. F. , N. L. Fowler , T. R. Petroelje , T. M. Kautz , D. E. Beyer , and J. L. Belant . 2022. “Ubms: An R Package for Fitting Hierarchical Occupancy and N‐Mixture Abundance Models in a Bayesian Framework.” Methods in Ecology and Evolution 13, no. 3: 577–584.

[ece371189-bib-0017] King, J. A. 1973. “The Ecology of Aggressive Behavior.” Annual Review of Ecology and Systematics 4: 117–138.

[ece371189-bib-0018] Leweri, C. M. , G. S. Bartzke , M. J. Msuha , and A. C. Treydte . 2022. “Spatial and Seasonal Group Size Variation of Wild Mammalian Herbivores in Multiple‐Use Landscapes of the Ngorongoro Conservation Area, Tanzania.” PLoS One 17, no. 4: e0267082. 35439256 10.1371/journal.pone.0267082 PMC9017940

[ece371189-bib-0019] Makin, D. F. , S. Chamaillé‐Jammes , and A. M. Shrader . 2017. “Herbivores Employ a Suite of Antipredator Behaviours to Minimize Risk From Ambush and Cursorial Predators.” Animal Behaviour 127: 225–231.

[ece371189-bib-1003] Menon, V. 2023. Indian Mammals: A Field Guide. Hachette India.

[ece371189-bib-1004] Nowak, R. M. 1999. Walker's Mammals of the World. JHU Press.

[ece371189-bib-0020] Olff, H. , M. E. Ritchie , and H. H. T. Prins . 2002. “Global Environmental Controls of Diversity in Large Herbivores.” Nature 415, no. 6874: 901–904. 10.1038/415901a. 11859367

[ece371189-bib-0021] Owen‐Smith, R. N. 1988. Megaherbivores: The Influence of Very Large Body Size on Ecology. Cambridge University Press.

[ece371189-bib-0022] Paliwal, A. , and V. B. Mathur . 2012. “Predicting Potential Distribution of Gaur (*Bos gaurus*) in Tadoba‐Andhari Tiger Reserve, Central India.” Journal of Life Sciences 6, no. 10: 1041.

[ece371189-bib-0023] Plummer, M. , N. Best , K. Cowles , and K. Vines . 2006. “{CODA}: Convergence Diagnosis and Output Analysis for {MCMC}.” R News 6, no. 1: 7–11.

[ece371189-bib-0024] Prayoon, U. , W. Suksavate , A. Chaiyes , et al. 2024. “Home Range and Habitat Utilization of Gaur (*Bos gaurus*) in the Transition Zone Between Protected Forest and Human‐Dominated Landscape, Eastern Thailand.” Global Ecology and Conservation 50: e02811.

[ece371189-bib-0025] Pringle, R. M. , J. O. Abraham , T. M. Anderson , et al. 2023. “Impacts of Large Herbivores on Terrestrial Ecosystems.” Current Biology 33, no. 13: R584–R610. 37279691 10.1016/j.cub.2023.04.024

[ece371189-bib-0026] QGIS Development Team . 2023. “QGIS Geographic Information System.” Open Source Geospatial Foundation Project.

[ece371189-bib-1005] R Core Team . 2023. R: A Language and Environment for Statistical Computing. R Foundation for Statistical Computing. Vienna, Austria. http://www.R‐project.org.

[ece371189-bib-0027] Regmi, S. , J. L. Belant , B. Pant , and H. P. Sharma . 2023. “Factors Influencing Mammalian Community Occupancy in Dhorpatan Hunting Reserve, Nepal.” Ecology and Evolution 13, no. 2: e9980. 37038514 10.1002/ece3.9980 PMC10082153

[ece371189-bib-0028] Rich, L. N. , D. A. W. Miller , H. S. Robinson , J. W. McNutt , and M. J. Kelly . 2016. “Using Camera Trapping and Hierarchical Occupancy Modeling to Evaluate the Spatial Ecology of an African Mammal Community.” Journal of Applied Ecology 53, no. 4: 1225–1235.

[ece371189-bib-0029] Ripple, W. J. , T. M. Newsome , C. Wolf , et al. 2015. “Collapse of the world's Largest Herbivores.” Science Advances 1, no. 4: e1400103. 10.1126/sciadv.1400103. 26601172 PMC4640652

[ece371189-bib-0030] Royle, J. A. , and R. M. Dorazio . 2008. Hierarchical Modeling and Inference in Ecology: The Analysis of Data From Populations, Metapopulations, and Communities. Elsevier.

[ece371189-bib-0031] Sankar, K. , H. S. Pabla , C. K. Patil , et al. 2013. “Home Range, Habitat Use and Food Habits of Re‐Introduced Gaur (*Bos gaurus gaurus*) in Bandhavgarh Tiger Reserve, Central India.” Tropical Conservation Science 6, no. 1: 50–69. 10.1177/194008291300600108.

[ece371189-bib-0033] Schmied née Stommel, C. , H. Hofer , C. Scherer , S. Kramer‐Schadt , and M. L. East . 2024. “Effect of Human‐Induced Surface Water Scarcity on Herbivore Distribution During the Dry Season in Ruaha National Park.” Tanzania. Wildlife Biology 2024: e01131.

[ece371189-bib-0034] Sharma, H. P. , B. P. Bhattarai , S. Regmi , et al. 2025. “Spatio‐Temporal Patterns of Tigers in Response to Prey Species and Anthropogenic Activities.” Proceedings B 292, no. 2039: 20241939. 10.1098/rspb.2024.1939 PMC11775626 39876738

[ece371189-bib-0035] Sharma, H. P. , H. B. Katuwal , B. P. Bhattarai , et al. 2023. “Factors Affecting the Occupancy of Sloth Bear and Its Detection Probability in Parsa–Koshi Complex, Nepal.” Ecology and Evolution 13, no. 5: e10587. 37794874 10.1002/ece3.10587 PMC10547580

[ece371189-bib-0036] Steinheim, G. , P. Wegge , J. I. Fjellstad , S. R. Jnawali , and R. B. Weladji . 2005. “Dry Season Diets and Habitat Use of Sympatric Asian Elephants ( *Elephas maximus* ) and Greater One‐Horned Rhinoceros (*Rhinocerus unicornis*) in Nepal.” Journal of Zoology 265, no. 4: 377–385.

[ece371189-bib-0037] Sunarto, S. , M. J. Kelly , K. Parakkasi , S. Klenzendorf , E. Septayuda , and H. Kurniawan . 2012. “Tigers Need Cover: Multi‐Scale Occupancy Study of the Big Cat in Sumatran Forest and Plantation Landscapes.” PLoS One 7, no. 1: e30859. 22292063 10.1371/journal.pone.0030859 PMC3264627

[ece371189-bib-0038] Tuomi, M. , S. Stark , K. S. Hoset , et al. 2019. “Herbivore Effects on Ecosystem Process Rates in a Low‐Productive System.” Ecosystems 22, no. 4: 827–843. 10.1007/s10021-018-0307-4.

[ece371189-bib-0039] Valeix, M. , S. Chamaillé‐Jammes , and H. Fritz . 2007. “Interference Competition and Temporal Niche Shifts: Elephants and Herbivore Communities at Waterholes.” Oecologia 153, no. 3: 739–748. 17566783 10.1007/s00442-007-0764-5

[ece371189-bib-0040] Wikramanayake, E. , E. Dinerstein , J. Seidensticker , et al. 2011. “A Landscape‐Based Conservation Strategy to Double the Wild Tiger Population.” Conservation Letters 4, no. 3: 219–227. 10.1111/j.1755-263x.2010.00162.x.

[ece371189-bib-0041] Zangmo, P. , D. B. Gurung , L. Letro , and S. Wangmo . 2018. “Distribution, Abundance and Occupancy of Gaur (*Bos gaurus* Smith) in the Royal Manas National Park, Bhutan.” Bhutan Journal of Natural Resources and Development 5, no. 1: 1–12. 10.17102/cnr.2018.01.

